# Gender-specific contribution of cardiometabolic index and lipid accumulation product to left ventricular geometry change in general population of rural China

**DOI:** 10.1186/s12872-018-0798-0

**Published:** 2018-04-10

**Authors:** Haoyu Wang, Yingxian Sun, Zhao Li, Xiaofan Guo, Shuang Chen, Ning Ye, Yichen Tian, Lijun Zhang

**Affiliations:** 1 Department of Cardiology, The First Hospital of China Medical University, 155 Nanjing North Street, Heping District, Shenyang, 110001 Liaoning People’s Republic of China; 2 Department of Hematology, The First Hospital of China Medical University, 155 Nanjing North Street, Heping District, Shenyang, 110001 Liaoning People’s Republic of China

**Keywords:** Left ventricular geometry, Left ventricular remodeling, Cardiometabolic index, Lipid accumulation product, Obesity, Adipose tissue, Gender-specific, Epidemiology

## Abstract

**Background:**

Despite current interest in the unfavorable impact of cardiometabolic index (CMI) and lipid accumulation product (LAP) on diabetes and cardiovascular risk, information regarding the relation of CMI and LAP to left ventricular (LV) geometry has not been specifically addressed. We aimed to examine the hypothesis: (1) CMI and LAP represent an independent determinant of LV remodeling in general population of rural China; (2) there are gender differences in obesity-related alterations in terms of LV morphology.

**Methods:**

The sample for this cross-sectional analysis included 11,258 participants (mean age 53.9 years; 54.0% females) who underwent assessment of basic metabolic and anthropometric parameters in rural areas of northeast China. Comprehensive echocardiography-defined LV geometric pattern was determined according to left ventricular mass index and relative wall thickness.

**Results:**

The prevalence rate of eccentric and concentric LV hypertrophy (LVH) presented a proportional increase with elevated quartiles of CMI and LAP in a dose-response manner (all *P* < 0.005). When CMI and LAP were entered as a continuous variable in multivariable adjusted model, we observed the independent effect of 1 SD increment in CMI and LAP with the probability of eccentric and concentric LVH, while this relationship was more pronounced in females than in males. Likewise, the odds ratio comparing the top versus bottom quartiles of CMI were 2.105 (95%CI:1.600–2.768) for eccentric LVH and 2.236 (95%CI:1.419–3.522) for concentric LVH in females. Males in the highest CMI quartile exhibited a nearly doubled (OR:1.724, 95%CI:1.287–2.311) and 1.523-fold (95%CI:1.003–2.313) greater risk of eccentric and concentric LVH, respectively. Increasing LAP entailed a higher possibility of eccentric LVH by a factor of 3.552 and 1.768 in females and males, respectively. In contrast to females, where LAP fourth quartile and concentric LVH were positively associated (OR:2.544, 95%CI:1.537–4.209), higher LAP did not correlate with concentric LVH in males (OR:1.234, 95%CI:0.824–1.849).

**Conclusions:**

CMI and LAP give rise to a new paradigm of accounting for gender difference in obesity-related abnormal LV geometry, an effect that was substantially greater in females. These two indices, acting in concert, may also be advantageous prognostically for refining cardiovascular risk stratification in individuals with LV remodeling.

**Electronic supplementary material:**

The online version of this article (10.1186/s12872-018-0798-0) contains supplementary material, which is available to authorized users.

## Background

Obesity is recognized as an important contributor to heart failure and cardiovascular disease (CVD) independent of comorbid illnesses [1–3]. The increase in body weight and composition was thought to be associated with the changes in left ventricular (LV) structure, attributable to increased hemodynamic load and a cluster of altered inflammatory and metabolic state [4–6]. It has been well documented that chronic volume load in response to obesity exhibited increased LV mass and cavity size, resulting in eccentric left ventricular hypertrophy (LVH) [7, 8]. Likewise, concentric LVH characterized by an increase in LV wall thickness (LVWT) greater than the chamber radius and elevated mass-to-volume ratio, was largely related to the impact of excess adiposity [4, 8–10]. Given that being the precursor for worsening cardiac function, LV structural abnormalities impose an elevated risk for progression of symptomatic heart failure and are considered as a powerful, integrated predictor of cardiovascular morbidity and mortality [11–13]. On this basis, it is now becoming evident that the effect of obesity on LV remodeling offers a conceivable pathophysiologic explanation for the relationship of excess body fat with adverse cardiovascular outcomes. Determining which measure of adiposity and obesity contributes to the changes in LV geometry could promote early identification and prevention of cardiovascular events.

There is a diverse geometric pattern of left ventricle adaptations to fat distribution, in view of adipose depots inherently involve disparate metabolic process, structural composition, and functional manifestation [14]. In this context, a sample of 1073 young individuals from Bogalusa Heart Study revealed that increases in body mass index (BMI), waist circumference (WC), and waist-to-height ratio (WHtR) were demonstrated to have independent influences on the development of eccentric LVH but not concentric LVH [15]. Interestingly, in a multiethnic cohort of 1262 adults from the Dallas Heart Study, the predominant impact of general or central adiposity on LV phenotype was concentric remodeling without additional ejection fraction change [16]. The notion that visceral adipose tissue, an index of central adiposity, was a key independent determinant of LV concentricity and unfavorable hemodynamics has been recently arisen from two observations [17, 18]. These findings placed particular emphasis on the potential role for adiposity and its distribution in LV morphology, accounting for the pathogenic correlates of future heart failure.

Lipid accumulation product (LAP) is an emerging central lipid accumulation parameter in the light of taking both waist circumference (WC) and triglyceride (TG) levels into account, with prior studies reporting its strong predictive of clinical cardiovascular consequences and all-cause mortality [19, 20]. Lately, a novel parameter, named cardiometabolic index (CMI), has been put forward by Ichiro Wakabayashi to adequately discriminate the presence of diabetes and atherosclerotic progression [21–23]. It represents a combination of TG/ high-density lipoprotein cholesterol (HDL-C) ratio and WHtR, highlighting the product of them integrates the blood lipid and abdominal obesity into a clinically accessible and conceptually appealing marker. Of note, CMI could be clinically relevant with respect to the LV geometry, with insulin resistance and carotid intima-media thickness (IMT) having a substantial effect on cardiac remodeling [24, 25]. However, none of the studies that address the impact of measures of adiposity (CMI and LAP) on the pathogenesis of abnormal LV geometry has been specifically established. In effect, the gender difference in fat quantity, anatomic storage and consequent hemodynamic influence has been proposed to drive a better understanding of the link between obesity and LV remodeling. Accordingly, the aim of this study was to provide further insight into exploring CMI and LAP as a reliable biomarker for gender-specific identifying those subjects at higher risk for LV geometry in general population of rural China.

## Methods

### Study population

The data originated from a large population-based epidemiological cross-sectional study of 11,956 permanent residents (≥35 years of age) in rural areas of China. The full details regarding the study design and definitions were extensively described elsewhere [26–28]. After 698 of subjects were excluded because of missing biochemical and clinical covariates, our complete dataset (adiposity indices and echocardiographic data) consisted of 11,258 participants in the current analysis. The Ethics Committee of China Medical University (Shenyang, China) approved the study protocol. We obtained written informed consent from each participant before enrollment, and the whole data and procedures conformed to the principles of ethical standards.

### Data collection and measurements

The detailed process about data collection and methods selection of this sample has been fully reported in our prior publications [26–28]. Cardiologists and trained nurses administered a structured questionnaire to document specified data on demographic, health-related behaviors, anthropometric parameters, history of CVD (coronary heart disease, arrhythmia and heart failure), use of antihypertensive drug (at least one type versus no), use of antidiabetic drug (at least one type versus no), and use of lipid-lowering drug (at least one type versus no).

Study participants waited for at least 5 min in a relaxed and sitting position. Then blood pressure was measured by two randomly trained observers. The three consecutive readings were collected on the right arm at 1–2 min intervals and the average value was recorded for analysis.

Anthropometric indices were conducted when the subjects wore in light clothing without shoes. Their weight was quantified to the nearest 0.1 kg in the utility of a calibrated digital scale. A portable stadiometer was used for height measurement (rounded to nearest 0.1 cm) in a standing position. After full expiration, WC was measured using a steel measuring tape from the horizontal line at 1 cm above the belly button. Anthropometric indices were measured twice and then averaged. BMI was calculated as weight per height squared (kg/m^2^). WHtR was defined as WC divided by height in meters squared.

Fasting antecubital vein blood specimens were designed to be obtained by each participant in the morning after an overnight fasting with 12 h for assessing fasting plasma glucose (FPG), TG, and HDL-C. Comprehensive storage process and laboratory measurement methods were available in the previous reports [26–28]. The TG/HDL-C ratio was calculated using available TG and HDL-C values.

### Echocardiography

Information on the echocardiography examination was conducted according to standardized procedures, as previously described [28, 29]. Two-dimensional, M-mode, spectral and color Doppler transthoracic echocardiography were performed with a commercially available Doppler machine (Vivid, GE Healthcare, United States) and were interpreted by three skilled echocardiographers.

Measurements of LV end-diastolic internal dimension (LVIDD), interventricular septal thickness (IVST), and posterior wall thickness (PWT) were determined by the optimized parasternal long-axis view on the basis of the recommendation from the American Society of Echocardiography [30]. Using the Teichholz equations [31], we assessed the LV end-diastolic volume (LVEDV), LV end-systolic volume (LVESV) to generate LV ejection fraction. Left ventricular mass (LVM) was derived from a necropsy validated formula: LVM = 0.8× [1.04{(IVST + PWT + LVIDD)^3^–LVIDD^3^}] + 0.6 g [32]. Normalization of LVM for height to the power of 2.7 was regarded as left ventricular mass index (LVMI). LVWT = (IVST + PWT) / 2. The relative wall thickness (RWT) was calculated as 2 × PWT/ LVIDD. The presence of LV hypertrophy was categorized by gender-specific cutoffs of LVMI > 46.7 g/m^2.7^ for females and > 49.2 g/m^2.7^ for males. Concentric LV geometry was defined as a partition value of 0.42 for RWT in both females and males [33]. Four different pattern of LV geometry were defined: (1) normal geometry was confirmed when the values of LVMI and RWT were within normal range; (2) concentric LV remodeling with normal LVMI and increased RWT; (3) increased LVMI and normal RWT was classified as eccentric LVH; and (4) concentric LVH with increases in both LVMI and RWT.

### Definitions

CMI was calculated according to the formula: CMI = TG/HDL-C × WHtR. Lipid accumulation product (LAP) is determined by using the following equation: LAP = TG (mmol/l) × (WC (cm) − 58) for women and LAP = TG (mmol/l) × (WC (cm) − 65) for men.

### Statistical analyses

Analyses were conducted separately for each gender. Baseline characteristics of all individuals are summarized as mean ± standard deviation or median (interquartile range) and numbers (percentage) as appropriate. Logarithmic transformation was performed for CMI and LAP because of skewness. Characteristics were assessed females and males using Student’s t or Mann-Whitney test to examine differences in means, while χ2 test for independence was utilized to compare differences of categorical variables in proportions. Baseline demographic, clinical, biochemical, and anthropometric measures and echocardiogram parameters were compared across four categories of LV geometry using one-way ANOVA or Kruskal-Wallis test for continuous variables and χ2 test for categorical data. The strength of linear association between left ventricular geometry parameters and anthropometric indices was tested by Spearman correlation coefficients. CMI and LAP were stratified into quartiles in accordance with the distribution of each anthropometric measure. We obtained gender-specific estimation of the OR for 1 SD increment in CMI and LAP to predict the risk of LV geometric abnormalities. Additionally, stratified by gender, multivariable logistic regression analysis was applied to explore an independent association of quartiles of CMI and LAP on the risk of eccentric LVH and concentric LVH, where three models were evaluated when potential confounding risk factors were progressively established. The results are expressed as odds ratios (ORs) and 95% confidence intervals (CIs). All of the statistical analyses involved the application of SPSS 22.0 software (IBM Corp) and a two-tailed *P* < 0.05 was adopted to be statistically significant.

## Results

### Demographic, clinical, and echocardiographic characteristics of study participants stratified by gender

The comparison between females (*n* = 6079) and males (*n* = 5179) with regard to the demographic, clinical, and echocardiographic characteristics were shown in Table 1. The mean age of the study subjects was 53.86 ± 10.54 years, and the average age of males was slightly higher than females. Overall, the metabolic parameters like systolic blood pressure (SBP), diastolic blood pressure (DBP), FPG, WHtR, and WC are prone to higher in males than females (all *P* < 0.05). No significant differences were presented between the groups for HDL-C, TG/HDL-C and diabetes. Relative to males, females had greater TG, CMI, and LAP, with a more frequency in use of antihypertensive drug, antidiabetic drug, and history of coronary heart disease and arrhythmia. For descriptive results of echocardiographic indices related to LV structure, females were associated with lower LVIDD, IVST, PWT, LVWT, LVM, LVMI, LVEDV, LVESV in comparison to males, without a significant distinction in LV end-systolic volume index (LVESVI). As expected, increased RWT, LV end-diastolic volume index (LVEDVI), and LV ejection fraction were found in females. The proportion of concentric remodeling and eccentric LVH were somewhat higher in females apart from concentric LVH which was quite comparable in both genders.

**Table 1:** Demographic, clinical, and echocardiographic characteristics of study participants stratified by gender

Variable	Total (*N* = 11,258)	Female (N = 6079)	Male (N = 5179)	*P* value*
Age (years)	53.86 ± 10.54	53.39 ± 10.32	54.42 ± 10.78	< 0.001
Race (Han) (%)	10,681 (94.9)	5769 (94.9)	4912 (94.8)	0.893
Primary school or below (%)	5629 (50.0)	3459 (56.9)	2710 (41.9)	< 0.001
Family income > 20,000 CNY/year (%)	3709 (32.9)	2014 (33.1)	1695 (32.7)	0.008
Low physical activity (%)	3336 (29.6)	2171 (35.7)	1165 (22.5)	< 0.001
Diet score	2.32 ± 1.13	2.13 ± 1.11	2.54 ± 1.10	< 0.001
Current smoker (%)	3959 (35.2)	996 (16.4)	2963 (57.2)	< 0.001
Current drinker (%)	2523 (22.4)	177 (2.9)	2346 (45.3)	< 0.001
Systolic blood pressure (mmHg)	141.69 ± 23.40	140.09 ± 24.02	143.57 ± 22.51	< 0.001
Diastolic blood pressure (mmHg)	82.04 ± 11.75	80.57 ± 11.53	83.76 ± 11.78	< 0.001
Fasting plasma glucose (mmol/L)	5.91 ± 1.63	5.87 ± 1.61	5.95 ± 1.67	0.007
TG (mmol/L)	1.24 (0.88–1.89)	1.26 (0.90–1.91)	1.23 (0.86–1.88)	0.017
HDL-C (mmol/L)	1.41 ± 0.38	1.41 ± 0.34	1.41 ± 0.42	0.717
TG/HDL-C ratio	0.92 (0.59–1.54)	0.93 (0.60–1.53)	0.91 (0.58–1.57)	0.566
WC (cm)	82.43 ± 9.82	81.29 ± 9.73	83.76 ± 9.76	< 0.001
Height (m)	1.61 ± 0.08	1.56 ± 0.06	1.66 ± 0.06	< 0.001
WHtR	0.51 ± 0.06	0.50 ± 0.06	0.52 ± 0.06	< 0.001
Hypertension (%)	5738 (51.0)	2949 (48.5)	2789 (53.9)	< 0.001
Diabetes (%)	1175 (10.4)	662 (10.9)	513 (9.9)	0.089
Antihypertensive drug (%)^a^	1701 (15.1)	1034 (17.0)	667 (12.9)	< 0.001
Antidiabetic drug (%)^a^	449 (4.0)	294 (4.8)	155 (3.0)	< 0.001
Lipid-lowering drug (%)^a^	371 (3.3)	211 (3.5)	160 (3.1)	0.258
History of CVD				
Coronary heart disease (%)	580 (5.2)	363 (6.0)	217 (4.2)	< 0.001
Arrhythmia (%)	617 (5.5)	421 (6.9)	196 (3.8)	< 0.001
Heart failure (%)	104 (0.9)	58 (1.0)	46 (0.9)	0.716
CMI	0.47 (0.29–0.82)	0.48 (0.30–0.83)	0.46 (0.27–0.82)	0.002
LAP (cm·mmol/L)	25.44 (13.39–47.38)	28.31 (16.17–50.95)	21.95 (10.71–43.60)	< 0.001
ECG measures				
LVIDD (cm)	4.74 ± 0.46	4.54 ± 0.41	4.89 ± 0.45	< 0.001
IVST (cm)	0.89 ± 0.28	0.86 ± 0.26	0.92 ± 0.30	< 0.001
PWT (cm)	0.87 ± 0.31	0.85 ± 0.33	0.90 ± 0.29	< 0.001
LVWT (cm)	2.79 ± 0.28	2.69 ± 0.25	2.90 ± 0.27	< 0.001
RWT	0.38 ± 0.33	0.39 ± 0.41	0.37 ± 0.20	0.050
LVM (g)	132.32 (113.63–158.21)	122.26 (105.33–137.72)	147.78 (128.02–174.52)	< 0.001
LVMI (g/m^2.7^)	36.99 (31.98–43.43)	36.69 (31.52–42.84)	37.40 (32.46–44.13)	< 0.001
LVEDV (ml)	104.17 ± 22.95	95.87 ± 19.17	113.94 ± 23.18	< 0.001
LVESV (ml)	39.45 ± 13.60	36.16 ± 11.68	43.34 ± 14.64	< 0.001
LVEDVI (ml/m^2.7^)	29.07 ± 6.35	29.18 ± 6.29	28.93 ± 6.42	0.037
LVESVI (ml/m^2.7^)	11.01 ± 3.76	11.00 ± 3.65	11.01 ± 3.88	0.902
LV ejection fraction (%)	62.05 ± 10.02	62.22 ± 9.76	61.85 ± 10.31	0.048
Normal geometry	8780 (78.0)	4656 (76.6)	4124 (79.6)	< 0.001
Concentric LV remodeling	792 (7.0)	466 (7.7)	326 (6.3)	0.005
Eccentric LVH	1185 (10.5)	689 (11.3)	496 (9.6)	0.002
Concentric LVH	501 (4.5)	268 (4.4)	233 (4.5)	0.817

Data are expressed as mean ± standard deviation or median (interquartile range) and numbers (percentage) as appropriate. *CNY* China Yuan (1CNY = 0.158 USD), *TG* triglyceride, *HDL-C* high-density lipoprotein cholesterol, *WHtR* waist-to-height ratio, *CVD* cardiovascular disease, *CMI* cardiometabolic index, *LAP* lipid accumulation product, *LV* left ventricular, *IVST* interventricular septal thickness, *LVIDD* left ventricular end-diastolic internal dimension, *PWT* posterior wall thickness, *LVWT, LV* wall thickness, *RWT*, relative wall thickness, *LVM* left ventricular mass, *LVMI* left ventricular mass index, *LVEDV* left ventricular end-diastolic volume, *LVESV* left ventricular end-systolic volume LVEDVI, left ventricular end-diastolic volume index, *LVESVI* left ventricular end-systolic volume index, *ECG* echocardiogram, *LVH* left ventricular hypertrophy

^*^ *P* values are for a Student’s t or Mann-Whitney test (continuous) and chi-square test (categorical) comparison across left ventricular geometry

^a^ At least one, versus no

Characteristics of study population according to the left ventricular geometry were listed in Additional file 1: Table S1. Of 11,258 adults, 8780 (78.0%) presented with normal LV geometry, 792 (7.0%) presented with concentric remodeling, 1185 (10.5%) presented with eccentric LVH, and 501 (4.5%) presented with concentric LVH. The level of SBP, DBP, FPG, TG, and TG/HDL-C tended to increase in proportion to LV structure from normal LV geometry to concentric LVH. In anthropometric measures of CMI and LAP, participants with either eccentric or concentric LVH showed the more unfavorable mean value than normal LV geometry. Especially, use of antihypertensive drug, and antidiabetic drug, lipid-lowering drug and history of CVD (coronary heart disease, arrhythmia and heart failure) were more prevalent in subjects with LVH, regardless of the geometric pattern, than in adults with normal LV geometry.

### Spearman’s correlation analysis of CMI and LAP with left ventricular geometry parameters by gender

Table 2 indicated the unadjusted Spearman’s correlation coefficients regarding the association between measures of obesity and LV geometry by different genders. Increasing of CMI and LAP were highly related to LVM, LVMI, LVIDD, LVEDVI, LVESVI, LVWT and RWT for both sexes, and the strength of the correlations was more pronounced among females (all *P* < 0.05). Put together, subjects showed a greater LV geometry parameter response to ascending LAP, a pattern that was more prominent than CMI.

**Table 2:** Spearman’s correlation analysis of CMI and LAP with left ventricular geometry parameters by gender

	LVM	LVMI	LVIDD	LVEDVI	LVESVI	LVWT	RWT
Female							
CMI	0.276^a^	0.282^a^	0.154^a^	0.181^a^	0.139^a^	0.204^a^	0.130^a^
LAP	0.382^a^	0.341^a^	0.238^a^	0.217^a^	0.167^a^	0.300^a^	0.141^a^
Male							
CMI	0.195^a^	0.159^a^	0.118^a^	0.080^a^	0.039^b^	0.150^a^	0.074^a^
LAP	0.289^a^	0.207^a^	0.207^a^	0.117^a^	0.064^a^	0.243^a^	0.058^a^

Abbreviations: *LVM* left ventricular mass, *LVMI* left ventricular mass index, *LVIDD* left ventricular end-diastolic internal dimension, *LVEDVI* left ventricular end-diastolic volume index, *LVESVI* left ventricular end-systolic volume index, *LVWT, LV* wall thickness, *RWT* relative wall thickness, *CMI* cardiometabolic index, *LAP* lipid accumulation product

^a^ *P* < 0.01; ^b^ *P* < 0.05;

### Odds ratio (OR) and 95% confidence intervals for abnormal left ventricular geometry according to gender-specific continuous or quartiles of CMI and LAP

The multivariable logistic regression analysis was carried out to evaluate the gender-specific association of CMI and LAP with eccentric and concentric LVH (Table 3). In analyses modeling CMI and LAP as a continuous variable, we revealed a 32% and 50% higher risk for eccentric LVH with each SD increment in CMI and LAP levels in females, respectively. Similar results were acquired for the effect of 1 SD increase in CMI and LAP with eccentric LVH in males (OR, 1.187; 95% CI, 1.075–1.311; OR, 1.251; 95% CI, 1.123–1.394, respectively; model 3), while this association was particularly pronounced among females. Likewise, CMI carried concentric LVH odds (95% CIs) of 1.280 (1.122 to 1.461) and 1.172 (1.019 to 1.347) in females and males, respectively, per SD increase. The relationship between LAP and concentric LVH was significant with higher risks in the former (Females, OR, 1.384; 95% CI, 1.191–1.609; Males, OR, 1.233; 95% CI, 1.056–1.441). In models incorporating CMI and LAP quartiles, after taking into account the effect of confounding factors (model 2), participants in the highest quartile of CMI and LAP exhibited a 2.74-fold (95%CI, 2.13 to 3.61) and 4.99-fold (95% CI, 3.68 to 6.77) elevated risk of eccentric LVH compared with the lowest quartile in females. Similarly, the adjusted ORs of concentric LVH were statistically significant in the top quartile of CMI (OR, 3.610; 95% CI, 2.330 to 3.593) and LAP (OR, 4.904; 95% CI, 3.206 to 7.949). Adding SBP, DBP, FPG, use of antihypertensive drug, and antidiabetic drug, lipid-lowering drug and history of CVD (coronary heart disease, arrhythmia and heart failure) to the model 3 did not materially alter the results and the associations maintained statistical significance. As a result of this, it was consistent in females that higher CMI and LAP levels were independently and positively related to the probability of eccentric or concentric LVH in a dose-response pattern (all P for trend < 0.001). In contrast to females, when the highest and lowest categories were compared, a high LAP level demonstrated a 1.77-fold increase in the odds of eccentric LVH in males (95% CI, 1.305 to 2.396; P for trend< 0.001), whereas there was no relationship between concentric LVH and increasing LAP (P for trend = 0.120). Additionally, ORs of eccentric and concentric LVH increased by a factor of 1.72 and 1.52 for males with rising levels of CMI, respectively. In general, the associations of CMI and LAP with eccentric LVH or concentric LVH were more prominent in females.

**Table 3:** Odds ratio (OR) and 95% confidence intervals for abnormal left ventricular geometry according to gender-specific continuous or quartiles of CMI and LAP

Variables	Eccentric LVH				Concentric LVH			
		OR (95% CI)				OR (95% CI)		
	Case (%)	Model 1	Model 2	Model 3	Case (%)	Model 1	Model 2	Model 3
Females								
CMI (per 1 SD increase)		1.601 (1.482–1.730)	1.453 (1.339–1.576)	1.319 (1.209–1.438)		1.683 (1.500–1.888)	1.527 (1.352–1.723)	1.280 (1.122–1.461)
Quartiles of CMI								
Q1 (≤0.30)	84 (12.2)	1.000 (reference)	1.000 (reference)	1.000 (reference)	26 (9.7)	1.000 (reference)	1.000 (reference)	1.000 (reference)
Q2 (0.30–0.48)	117 (17.0)	1.491 (1.116–1.992)	1.341 (0.998–1.802)	1.258 (0.934–1.695)	45 (16.8)	1.829 (1.123–2.981)	1.609 (0.983–2.634)	1.451 (0.881–2.389)
Q3 (0.48–0.83)	222 (32.2)	2.938 (2.261–3.817)	2.407 (1.841–3.146)	2.063 (1.572–2.709)	77 (28.7)	3.083 (1.965–4.837)	2.443 (1.548–3.854)	1.806 (1.135–2.874)
Q4 (> 0.83)	266 (38.6)	3.736 (2.890–4.829)	2.742 (2.106–3.572)	2.105 (1.600–2.768)	120 (44.8)	5.065 (3.294–7.787)	3.610 (2.330–5.593)	2.236 (1.419–3.522)
*P* value for trend	< 0.001	< 0.001	< 0.001	< 0.001	< 0.001	< 0.001	< 0.001	< 0.001
LAP (per 1 SD increase)		1.874 (1.716–2.047)	1.668 (1.541–1.849)	1.496 (1.357–1.650)		1.955 (1.713–2.232)	1.742 (1.518–1.998)	1.384 (1.191–1.609)
Quartiles of LAP								
Q1 (≤16.17)	54 (7.9)	1.000 (reference)	1.000 (reference)	1.000 (reference)	20 (7.5)	1.000 (reference)	1.000 (reference)	1.000 (reference)
Q2 (16.17–28.31)	134 (19.5)	2.587 (1.870–3.579)	2.356 (1.695–3.274)	2.112 (1.515–2.946)	40 (15.0)	1.999 (1.163–3.435)	1.756 (1.017–3.031)	1.395 (0.802–2.427)
Q3 (28.31–50.95)	199 (29.0)	4.034 (2.959–5.499)	3.329 (2.429–4.563)	2.713 (1.970–3.735)	77 (28.9)	3.949 (2.402–6.492)	3.095 (1.873–5.114)	2.108 (1.263–3.517)
Q4 (> 50.95)	300 (43.7)	6.580 (4.877–8.879)	4.957 (3.653–6.727)	3.552 (2.587–4.876)	129 (48.5)	6.858 (4.258–11.046)	4.904 (3.206–7.949)	2.544 (1.537–4.209)
*P* value for trend	< 0.001	< 0.001	< 0.001	< 0.001	< 0.001	< 0.001	< 0.001	< 0.001
Males								
CMI (per 1 SD increase)		1.264 (1.156–1.383)	1.327 (1.208–1.458)	1.187 (1.075–1.311)		1.309 (1.155–1.483)	1.389 (1.221–1.579)	1.172 (1.019–1.347)
Quartiles of CMI								
Q1 (≤0.27)	85 (17.1)	1.000 (reference)	1.000 (reference)	1.000 (reference)	39 (16.7)	1.000 (reference)	1.000 (reference)	1.000 (reference)
Q2 (0.27–0.46)	108 (21.8)	1.219 (0.908–1.638)	1.167 (0.865–1.575)	1.158 (0.855–1.567)	55 (23.6)	1.348 (0.888–2.047)	1.356 (0.890–2.065)	1.270 (0.826–1.954)
Q3 (0.46–0.82)	138 (27.8)	1.719 (1.296–2.280)	1.722 (1.292–2.295)	1.553 (1.160–2.080)	58 (24.9)	1.527 (1.010–2.309)	1.613 (1.063–2.448)	1.300 (0.846–1.997)
Q4 (> 0.82)	165 (33.3)	2.018 (1.534–2.654)	2.216 (1.672–2.935)	1.724 (1.287–2.311)	81 (34.8)	2.089 (1.414–3.085)	2.395 (1.611–3.562)	1.523 (1.003–2.313)
*P* value for trend	< 0.001	< 0.001	< 0.001	< 0.001	0.001	< 0.001	< 0.001	0.057
LAP (per 1 SD increase)		1.353 (1.228–1.490)	1.437 (1.297–1.591)	1.251 (1.123–1.394)		1.461 (1.273–1.677)	1.559 (1.351–1.793)	1.233 (1.056–1.441)
Quartiles of LAP								
Q1 (≤10.71)	77 (15.6)	1.000 (reference)	1.000 (reference)	1.000 (reference)	43 (18.5)	1.000 (reference)	1.000 (reference)	1.000 (reference)
Q2 (10.71–21.85)	98 (19.9)	1.242 (0.912–1.692)	1.200 (0.877–1.642)	1.092 (0.795–1.499)	41 (17.6)	0.914 (0.591–1.412)	0.900 (0.581–1.395)	0.711 (0.454–1.113)
Q3 (21.85–43.60)	153 (31.0)	2.031 (1.526–2.702)	2.035 (1.521–2.723)	1.682 (1.250–2.264)	60 (25.8)	1.357 (0.910–2.023)	1.396 (0.933–2.090)	0.940 (0.619–1.427)
Q4 (> 43.60)	165 (33.5)	2.215 (1.670–2.938)	2.489 (1.864–3.324)	1.768 (1.305–2.396)	89 (38.2)	2.063 (1.421–2.995)	2.345 (1.606–3.423)	1.234 (0.824–1.849)
*P* value for trend	< 0.001	< 0.001	< 0.001	< 0.001	< 0.001	< 0.001	< 0.001	0.091

Model 1: unadjusted; Model 2: adjusted for age, race, education level, family income, diet score, current smoking and drinking status, physical activity; Model 3: adjusted for all the factors in Model 2 and hypertension, diabetes, antihypertensive drug, and antidiabetic drug, lipid-lowering drug, and history of cardiovascular disease (coronary heart disease, arrhythmia and heart failure). Abbreviations: *OR* odd ratio; *95% CI* 95% confidence interval, *CMI* cardiometabolic index, *LAP* lipid accumulation product, *LVH* left ventricular hypertrophy

## Discussion

Novel findings in our study include an outstanding gender difference in the positive correlation of adiposity indices (CMI and LAP) with elevated likelihood for LV structural abnormality among the general Chinese population. The greater degree of eccentric and concentric LVH seen in obesity in response to increasing CMI and LAP is not only irrespective of conventional cardiovascular risk factors but is also pronounced in females than males, suggesting that excess adiposity per se influence abnormal LV geometry substantially more in females. Confirming CMI and LAP as a key independent determinant of LV hypertrophy and geometry had crucial implications for exploring potential areas of future investigations targeting CMI and LAP gender-specifically to prevent or weaken the effects of LV remodeling on heart disease.

Early data pointed out that due to a state of chronic volume overload, the prime effect of obesity on LV structure has been considered as eccentric LVH, with a resulting parallel increases in LV cavity size and LVWT and no alteration in RWT [7, 8, 34]. Instead, the results of recent studies consistently advocated a concentric pattern to be linked with increased weight, in nature with a greater extent increases in LVWT as compared to LVIDD accompanied by an increased RWT [4, 10, 35]. The majority of prior works evaluating the adverse impact of adiposity on LV geometry have mainly focused on parameters of general obesity such as BMI. The implication of BMI on eccentric LVH, represented by higher LVEDV, and concentric LVH geometric pattern, expressed by increased LVM/volume ratio, has been previously demonstrated in a cross-sectional fashion [4, 36]. A growing body of evidence supports the prognostic value of long-term change in BMI on certain aspects of LV geometry. Two researches from the Framingham Heart Study, performed in middle-aged adults, suggested that longitudinal tracking of LVWT, RWT and LV dimensions increased progressively with BMI over a 16 years period [37, 38]. Notably, in a Coronary Artery Risk Development in Young Adults (CARDIA) study, increasing BMI longitudinally over 25 years contributes to LV structural remodeling assessed by larger LV volume, greater LVM/height, and LVM/LVEDV ratio [39].

BMI has been the subject of numerous previous studies to interpret the influences of obesity on cardiovascular risk, while it was reported to be insusceptible to make a distinction between the excess adipose tissue and heavy muscle mass. It is now clear that body fat distribution, especially central adiposity, is believed to provide additional information than sheet quantity of body fat [40, 41]. The prospective cohort Atherosclerosis Risk in Communities study (ARIC) noted that greater metric of abdominal obesity, defined by WC, constituted an independent risk factor for subclinical abnormalities in LV structure [42]. In contrast to a recent study from Bogalusa Heart Study in which an eccentric LVH but not concentric LVH was predominant for WC [15], the Dallas Heart Study and a report from South African community proposed that WC raised considerably the possibility of concentric LV remodeling phenotype, which is prognostically worse due to a high risk for cardiovascular death [10, 16]. Furthermore, there have discrepant conclusions concerning the malefic health consequences of visceral adiposity index (VAI) as a valid estimate for the prediction of LV remodeling and geometry. Current investigations of LV morphology have elucidated that individuals with more visceral adiposity predisposed to greater LVM/volume ratio and LVWT concurrent with a decrease in LVEDV, which represented the summation of multiple diverse aspects of concentric LV remodeling [17, 18]. Conversely, Hu T and colleagues failed to support long-term increases, in both magnitude and velocity of VAI, as a marker associated with abnormal LV geometry [15]. Hence, it would be useful to embark on a novel and easily assessed marker of abdominal adiposity in clinical practice to stratify the risk of divergent cardiovascular structural and hemodynamic phenotypes given the paucity of efficient diastolic heart failure therapies.

It is noteworthy that LAP, which is computed from WC and fasting TG, has offered mechanistic insight into worse cardiometabolic profile given its independent relationship with incident cardiovascular events [19, 20, 43], yet is not quantified in the clinical setting of abnormal LV geometry. At present, CMI, a product of TG/HDL-C ratio and WHtR, has the advantage of being applicable in the assessment of diabetes and atherosclerotic progression [21–23]. Ichiro Wakabayashi et al. reflected that contribution of elevated levels of CMI to the prevalent diabetes risk was somewhat stronger in females than in males [22]. A relevant study further expanded current knowledge by confirming the importance of central adiposity (by CMI) as a potential etiology of atherosclerotic progression (by IMT) in subjects with peripheral arterial disease [21]. Given the fact that diabetes and atherosclerosis have been the primary mediators of subclinical LV remodeling, it is likely that CMI could explain the adverse cardiovascular effects of central obesity [24, 25]. In this sense, this middle-aged, cross-sectional, population-based design is initiated to examine and validate the practicality of CMI and LAP as the key correlates of LV geometric abnormalities in rural China.

Our data underlined the clinical value of new central adiposity measures such as LAP and CMI in assessing the risk of LVH which differed somewhat by gender. There was a greater detrimental influence of CMI on abnormal LV morphology in females compared with males. Our findings were coherent with those of previous clinical studies in that obesity was proved to elicit a cluster of LV geometric abnormalities, which is especially evident in females. A community-based sample of the MONICA Augsburg cohort described that the rise of LVH prevalence in response to obesity and hypertension was generally higher in females [44]. In keeping with this, a large, biracial cohort of elderly participants speculated that obese females, but not males, were significantly accompanied by abnormal LV geometry [42]. In the 2919 members of the Strong Heart Study cohort, increased LV mass was a function of ascending waist-to-hip ratio, a typical index reported in the context of central obesity [45]. There was a further demonstration that females, in whom adipose tissue was much more abundant, denoted excess LV mass substantially greater than males. In our study, the statistical effect of visceral distribution on the variability of LV mass in females was indicated by the significant impact of CMI and LAP. At each BMI-level, the greater degree of adipose tissue in females provides an explanation for this differential response [46]. A considerable proportion of females possess a higher percentage of body fat than males, which may in turn expose them to central obesity [47]. In addition, as the average age of females in our study was 53.39 years where sex hormone levels have declined, the favorable cardiovascular effects of estrogen were inevitably disappeared. Owing to the fact that estrogen is believed to inhibit cardiac hypertrophy and testosterone promotes LV hypertrophy, endogenous sex hormone differences might account for gender difference in obesity-related LV remodeling [48]. On the other hand, central obesity can cause dramatic changes in the release of inflammatory markers [49], which is the major driving force for regulating cardiac energy metabolism [50]. It is well known that as for normotensive obese females, pro-inflammatory cytokines have been implicated in both echocardiographic abnormalities and the amount of visceral adipose tissue [51]. Obesity and female gender interacted in determining myocardial glucose uptake and insulin sensitivity, which demonstrated gender-related differences in the myocardial substrate metabolism due to obesity [52].

There has been a great interest in exploring the underlying mechanism regarding the potential adverse impact of CMI and LAP on morphologic LV abnormalities. The cardiac workload in visceral obesity is constantly increased, probably as a consequence of supplying the high energy demands of the adipose tissue [53, 54]. An increase in LVM might be the result of chronic volume overload and insufficient adaptation of peripheral resistance to the increased cardiac output, characterizing a state of increased stroke work [54]. Secondly, central fat distribution, which has been assumed to be the metabolically active compartment of fat deposits, could mediate increased LVM through the effects of expression of circulating inflammatory cytokines, elevated availability of angiotensinogen, and increased myocardial fibrosis [53, 55]. Pro-inflammatory visceral adipocytes generate a cascade of neuro-hormonal signals, which play a critical role in insulin resistance and cardiac remodeling [56, 57]. Also, the crucial importance of hyperinsulinemia and insulin resistance in favoring myocardial hypertrophy is well recognized in the context of growth-stimulating effect of insulin or expansion of blood volume [58]. Thirdly, exposure of the heart to deposition of fat tissue and the presence of high fatty acid and TG levels in the myocardium impair cardiac structure and induce an increase in LVM [58, 59]. Moreover, superimposing higher systolic blood pressures to obesity combines hemodynamic (pressure, volume overload) and non-hemodynamic stimuli (fat infiltration, inflammation), which together has an additive effect on concentric myocardial remodeling [60].

Our results from this study are subject to some limitations. First, the cross-sectional design allows for only determining an association of CMI and LAP with abnormal LV geometry, but no potential cause-effect relationships can be drawn. Further confirmations in prospective studies to assess the prognostic role of CMI and LAP in LV morphology are warranted. Secondly, more detailed and precise imaging phenotypes of adiposity tissue distribution such as visceral or abdominal subcutaneous fat are not available in our study. Thirdly, our sample is comprised of only Chinese adults, and it is unknown whether our findings are also applicable to other racial or ethnic populations. Notwithstanding these limitations, the potential public health implications for the prevention and treatment of LVH and heart failure also merit comment. Our population-based design (which permits extensive multivariable adjustment for several confounders) for the first time acknowledges that CMI and LAP have a fulfilling supplementary beneficial effect on predicting LV geometric pattern in a large sample of community members of Northeast China. Within the scope of the epidemiological study, CMI and LAP are much more cost-effective and clinically feasible parameter to evaluate obesity-related pathologic cardiac remodeling when compared with quantitative measurements of visceral fat from advanced imaging. Under this scenario, cardiovascular risk stratification might be improved by adoption of simple central adiposity measures (defined by CMI and LAP).

## Conclusion

Females with greater CMI and LAP were more likely to lead in eccentric and concentric LVH with a concurrent decrement in their associations among males. Given the gender differences in LV adaptation to adiposity exists, our results add further impetus to translating CMI and LAP into a clinical and public health recommendations for reducing the burden of LVH. Efforts to target individuals at higher cardiometabolic risk on a gender-specific basis, integrating assessments of CMI and LAP may prevent abnormal LV geometry and incident heart failure.

## Additional file

Additional file 1: **Table S1**. Characteristics of study population according to the left ventricular geometry. (DOCX 18 kb)

## Abbreviations

BMI: Body mass index
CMI: Cardiometabolic index
CVD: Cardiovascular disease
DBP: Diastolic blood pressure
ECG: Echocardiogram
FPG: Fasting plasma glucose
HDL-C: High-density lipoprotein cholesterol
IVST: Interventricular septal thickness
LAP: Lipid accumulation product
LV: Left ventricular
LVEDV: Left ventricular end-diastolic volume
LVEDVI: Left ventricular end-diastolic volume index
LVESV: Left ventricular end-systolic volume
LVESVI: Left ventricular end-systolic volume index
LVH: Left ventricular hypertrophy
LVIDD: Left ventricular end-diastolic internal dimension
LVM: Left ventricular mass
LVMI: Left ventricular mass index
LVWT: LV wall thickness
PWT: Posterior wall thickness
RWT: Relative wall thickness
SBP: Systolic blood pressure
TG: Triglyceride
VAI: Visceral adiposity index
WC: Waist circumference
WHtR: Waist-to-height ratio
