# Supplementary material for: Gender-specific contribution of cardiometabolic index and lipid accumulation product to left ventricular geometry change in general population of rural China
Source: BMC Cardiovasc Disord. 2018 Apr 10;18:62. doi: 10.1186/s12872-018-0798-0 (PMC5891949; doi:10.1186/s12872-018-0798-0)
Supplement: Supplementary file 1 — Table S1. Characteristics of study population according to the left ventricular geometry. (DOCX 18 kb) [file 12872_2018_798_MOESM1_ESM.docx]

Additional file 1: Table S1 Characteristics of study population according to the left ventricular geometry

| Variable | Normal geometry (N=8780) | Concentric LV remodeling (N=792) | Eccentric LVH  (N=1185) | Concentric LVH  (N=501) | P value* |
| --- | --- | --- | --- | --- | --- |
| Age (years) | 52.48 ± 10.11 | 57.23 ± 11.01 | 59.44 ± 10.19 | 59.60 ± 10.54 | <0.001 |
| Female (%) | 4656 (53.0) | 466 (58.8) | 689 (58.1) | 268 (53.5) | <0.001 |
| Race (Han) (%) | 8315 (94.7) | 757 (95.6) | 1138 (96.0) | 471 (94.0) | 0.146 |
| Primary school or below (%) | 4110 (46.8) | 436 (55.1) | 763 (64.4) | 320 (63.9) | <0.001 |
| Family income >20,000 CNY/year (%) | 3057 (34.8) | 214 (27.0) | 306 (25.8) | 132 (26.3) | <0.001 |
| Low physical activity (%) | 2405 (27.4) | 280 (35.4) | 454 (38.3) | 197 (39.3) | <0.001 |
| Diet score | 2.37 ± 1.12 | 2.23 ± 1.15 | 2.08 ± 1.12 | 2.18 ± 1.09 | <0.001 |
| Current smoker (%) | 3148 (35.9) | 270 (34.1) | 360 (30.4) | 181 (36.1) | 0.002 |
| Current drinker (%) | 2033 (23.2) | 154 (19.4) | 215 (18.1) | 121 (24.2) | <0.001 |
| Systolic blood pressure (mmHg) | 137.79 ± 20.81 | 146.05 ± 25.69 | 156.70 ± 25.15 | 167.69 ± 25.90 | <0.001 |
| Diastolic blood pressure (mmHg) | 80.72 ± 10.83 | 83.81 ± 12.68 | 86.39 ± 13.15 | 92.03 ± 14.26 | <0.001 |
| Fasting plasma glucose (mmol/L) | 5.83 ± 1.53 | 6.07 ± 1.90 | 6.16 ± 1.82 | 6.40 ± 2.17 | <0.001 |
| TG (mmol/L) | 1.20 (0.85-1.80) | 1.33 (0.94-2.04) | 1.47 (1.02-2.19) | 1.58 (1.07-2.48) | <0.001 |
| HDL-C (mmol/L) | 1.41 ± 0.38 | 1.44 ± 0.41 | 1.36 ± 0.36 | 1.37 ± 0.37 | <0.001 |
| TG/HDL-C ratio | 0.88 (0.57-1.47) | 0.97 (0.63-1.64) | 1.16 (0.71-1.90) | 1.24 (0.76-2.12) | <0.001 |
| WC (cm) | 81.63 ± 9.52 | 81.37 ± 9.98 | 86.99± 9.89 | 87.26 ± 10.27 | <0.001 |
| Height (m) | 1.61 ± 0.08 | 1.59 ± 0.08 | 1.57 ± 0.09 | 1.58 ± 0.09 | <0.001 |
| WHtR | 0.51 ± 0.06 | 0.51 ± 0.06 | 0.56 ± 0.06 | 0.55 ± 0.07 | <0.001 |
| Hypertension (%) | 3882 (44.2) | 478 (60.4) | 927 (78.2) | 451 (90.0) | <0.001 |
| Diabetes (%) | 768 (8.7) | 108 (13.6) | 198 (16.7) | 101 (20.2) | <0.001 |
| Antihypertensive drug (%)^a^ | 931 (10.6) | 168 (21.2) | 368 (31.1) | 234 (46.7) | <0.001 |
| Antidiabetic drug (%)^a^ | 290 (3.3) | 42 (5.3) | 81 (6.8) | 36 (7.2) | <0.001 |
| Lipid-lowering drug (%)^a^ | 237 (2.7) | 20 (2.5) | 80 (6.8) | 34 (6.8) | <0.001 |
| History of CVD |  |  |  |  |  |
| Coronary heart disease (%) | 351 (4.0) | 53 (6.7) | 126 (10.6) | 50 (10.0) | <0.001 |
| Arrhythmia (%) | 440 (5.0) | 55 (6.9) | 85 (7.2) | 37 (7.4) | 0.001 |
| Heart failure (%) | 52 (0.6) | 10 (1.3) | 27 (2.3) | 15 (3.0) | <0.001 |
| CMI | 0.44 (0.27-0.76) | 0.49 (0.30-0.88) | 0.64 (0.39-1.08) | 0.67 (0.40-1.21) | <0.001 |
| LAP (cm·mmol/L) | 23.25 (12.48-43.05) | 27.14 (13.94-50.87) | 38.75 (21.60-64.15) | 40.88 (23.05-72.57) | <0.001 |
| **ECG measures** |  |  |  |  | <0.001 |
| LVIDD (cm) | 4.69 ± 0.36 | 4.12 ± 0.54 | 5.19 ± 0.50 | 4.71 ± 0.60 | <0.001 |
| IVST (cm) | 0.84 ± 0.08 | 0.95 ± 0.10 | 1.08 ± 0.65 | 1.19 ± 0.65 | <0.001 |
| PWT (cm) | 0.82 ± 0.07 | 0.94 ± 0.14 | 0.94 ± 0.10 | 1.43 ± 1.30 | <0.001 |
| LVWT (cm) | 2.76 ± 0.20 | 2.53 ± 0.29 | 3.06 ± 0.26 | 3.07 ± 0.62 | <0.001 |
| RWT | 0.35 (0.33-0.38) | 0.44 (0.43-0.46) | 0.37 (0.35-0.39) | 0.45 (0.43-0.49) | <0.001 |
| LVM (g) | 126.97 (109.69-145.22) | 118.67 (109.69-152.55) | 181.98 (158.21-213.18) | 200.50 (164.45-227.38) | <0.001 |
| LVMI (g/m^2.7^) | 35.29 (31.09-39.95) | 36.85 (31.78-41.82) | 53.04 (49.91-59.45) | 55.72 (51.86-65.04) | <0.001 |
| LVEDV (ml) | 102.75 ± 18.60 | 78.88 ± 14.72 | 129.85 ± 32.12 | 107.42 ± 20.13 | <0.001 |
| LVESV (ml) | 38.67 ± 12.13 | 31.24 ± 9.90 | 49.67 ± 19.06 | 41.69 ± 14.03 | <0.001 |
| LVEDVI (ml/m^2.7^) | 28.22 ± 4.55 | 22.60 ± 3.54 | 38.76 ± 9.17 | 31.12 ± 4.83 | <0.001 |
| LVESVI (ml/m^2.7^) | 10.62 ± 3.19 | 8.94 ± 2.64 | 14.82 ± 5.38 | 12.10 ± 3.92 | <0.001 |
| LV ejection fraction (%) | 62.31 ± 9.74 | 60.19 ± 11.08 | 61.66 ± 10.91 | 61.32 ± 10.46 | <0.001 |

Data are expressed as mean ± standard deviation or median (interquartile range) and numbers (percentage) as appropriate. CNY, China Yuan (1CNY = 0.158 USD); TG, triglyceride; HDL-C, high-density lipoprotein cholesterol; WHtR, waist-to-height ratio; CVD, cardiovascular disease; CMI, cardiometabolic index; LAP, lipid accumulation product; LV, left ventricular; IVST, interventricular septal thickness; LVIDD, left ventricular end-diastolic internal dimension; PWT, posterior wall thickness; LVWT, LV wall thickness; RWT, relative wall thickness; LVM, left ventricular mass; LVMI, left ventricular mass index; LVEDV, left ventricular end-diastolic volume; LVESV, left ventricular end-systolic volume LVEDVI, left ventricular end-diastolic volume index; LVESVI, left ventricular end-systolic volume index; ECG, echocardiogram; LVH, left ventricular hypertrophy.

* P values are for an ANOVA or Kruskal-Wallis test (continuous) and chi-square test (categorical) comparison across left ventricular geometry.

^a^ At least one, versus no
